# Estimation of Average Intraocular Lens Power for Cataract Surgeries in Trincomalee District, Sri Lanka: A Single-Centre Retrospective Descriptive Study

**DOI:** 10.7759/cureus.74583

**Published:** 2024-11-27

**Authors:** M Ninesh M D Pinto, DM Mangala Dissanayake, Kasturi Krishnamoorthy, Pavithra B Indrapala

**Affiliations:** 1 Ophthalmology, District General Hospital Trincomalee, Trincomalee, LKA

**Keywords:** cataract treatment, intraocular lens power, ophthalmology, phacoemulsification cataract surgery, sri lanka, trincomalee district

## Abstract

Introduction

The Sri Lankan economic crisis that began in 2019 led to the suspension of cataract services in many districts. Although humanitarian missions were quick to supply materials, there was a lack of scientific evidence to predict the required intraocular lens power for patients with cataracts. This study aimed to assess the average lens power among patients from Trincomalee district, Sri Lanka, based on sex and age groups.

Methods

Eye theatre records at District General Hospital Trincomalee were used to assess cataract surgeries between April 2022 and July 2023. Age, sex, side of intraocular lens insertion, and intraocular lens power (selected using ultrasound biometry and the SRK/T formula) were assessed.

Results

We assessed 1,045 patient records, comprising 476 male patients (46%) and 557 female patients (53%). The intraocular lenses used for the patients ranged from 3 to 30 diopters (D). The average lens power for the entire population was 22.95 D. It was significantly higher for the female patients (23.11 ± 2.519 D) compared with the male patients (22.75 ± 2.250 D) (p = 0.0168). Among the age groups, the highest prevalence of cataracts was in those aged 60-79 years.

Conclusions

Our findings provide a useful guide for surgical planning in a resource-constrained setting such as the Trincomalee district. While the prevalence of cataracts by age and sex is in accordance with global statistics, the average diopter size is slightly higher compared to Western, African, and other Asian populations.

## Introduction

An economic crisis in Sri Lanka began to unfold in 2019 and intensified markedly in 2020 and 2021 [1]. As stocks of essential medications decreased, physicians focused on procuring crucial drugs. In the past, Sri Lanka imported most of its medications, but due to the shortage of foreign currency, the chances of acquiring essential drugs decreased significantly. In April 2022, the cost of medications surged by 40% [2,3]. As a result of this economic crisis, the availability of intraocular lenses (IOLs) was drastically limited, leading to the suspension of cataract services in many areas of the country [4]. This disruption caused great distress among patients who had been waiting to undergo cataract surgery to improve their vision. While non-governmental organizations promptly addressed the shortage of IOLs, accurately predicting the necessary lens power remained a challenge.

Trincomalee district, located along the eastern coast of Sri Lanka, is home to an estimated 443,807 residents [5]. District General Hospital Trincomalee is a tertiary care center under the Central Ministry of Health of Sri Lanka. It serves as the main referral center for over 25 primary and secondary care institutions within the district. The Ophthalmology Department, led by a consultant general eye surgeon, is supported by a team of medical officers, resident house officers, optometrists, nurses trained in ophthalmic services, and healthcare assistants. The department is the main referral center for patients with eye-related issues from across Trincomalee district, providing both acute and non-acute inpatient and outpatient services. The General Eye Clinic sees patients three times a week, and there is a clinic for schoolchildren every Saturday. Cataract surgeries are performed on Tuesdays and Thursdays, with postoperative assessments conducted the day after surgery and again two weeks later.

In the present study, we aimed to determine the average lens power among patients who underwent cataract surgery in Sri Lanka's Trincomalee district. We also determined differences in lens power between male and female patients and several age groups. This endeavor should help establish a scientific basis for surgical planning and procurement strategies that can effectively manage similar supply constraints in the future.

## Materials and methods

Study design

This retrospective observational study was conducted solely at District General Hospital Trincomalee. We assessed patient records from the Ophthalmology Department over a 16-month period (April 2022 to July 2023).

Inclusion and exclusion criteria

The inclusion criteria were patients from Trincomalee district who had been assessed via the General Eye Clinic at District General Hospital Trincomalee and had received a posterior chamber intraocular lens (PCIOL) during the study period. The patients were selected for cataract surgery after assessment by the consultant ophthalmologist or an experienced medical officer. The preoperative assessment included ultrasound biometry performed by optometrists at the eye clinic to determine the appropriate IOL power, which was selected using the SRK/T formula. The exclusion criteria were traumatic cataracts, pediatric cataracts (in patients under 16 years of age), subluxated cataracts, cataracts in patients with macular edema (confirmed by optical coherence tomography), cataracts in patients with any form of retinal detachment, and cataracts in patients with macular diseases such as retinal vein occlusion, and age-related macular degeneration. These conditions could impact ultrasound biometry.

Data collection

We thoroughly examined the patient records from cataract surgeries conducted at the eye theatre of the General Eye Clinic at District General Hospital Trincomalee. We maintained patient confidentiality by anonymizing the records. We assessed a total of 1,045 records of patients who underwent PCIOL insertion. We analyzed the age, sex, and lens power of the patients. Of note, the records maintained in the eye theatre were paper-based, and product stickers containing unique lens details, such as IOL power, manufacturing information, and batch numbers, were affixed to the respective patient records. Due to illegible handwriting or missing documentation, likely resulting from human error, some records had to be excluded from the sex-related (12 patients) and age-related (three patients) analyses. The primary outcome was the average lens power. The secondary outcomes included the distribution of lens power between male and female patients and across five age groups.

Statistical analysis

We used descriptive and inferential statistics to analyze the data. We present the mean and standard deviation (SD) lens power for the entire cohort as well as separately for the male and female patients. We calculated the difference in the mean lens power between the male and female patients (as well as the 95% confidence interval) and used an independent-sample t-test to compare the mean lens power between the male and female patients. In addition, we divided the cohort into five age groups and performed analysis of variance (ANOVA) followed by post hoc analysis with Tukey’s honestly significant difference test to determine differences in the mean lens power between the age groups. We considered p < 0.05 to indicate a statistically significant difference.

## Results

Lens power

**Figure 1:**
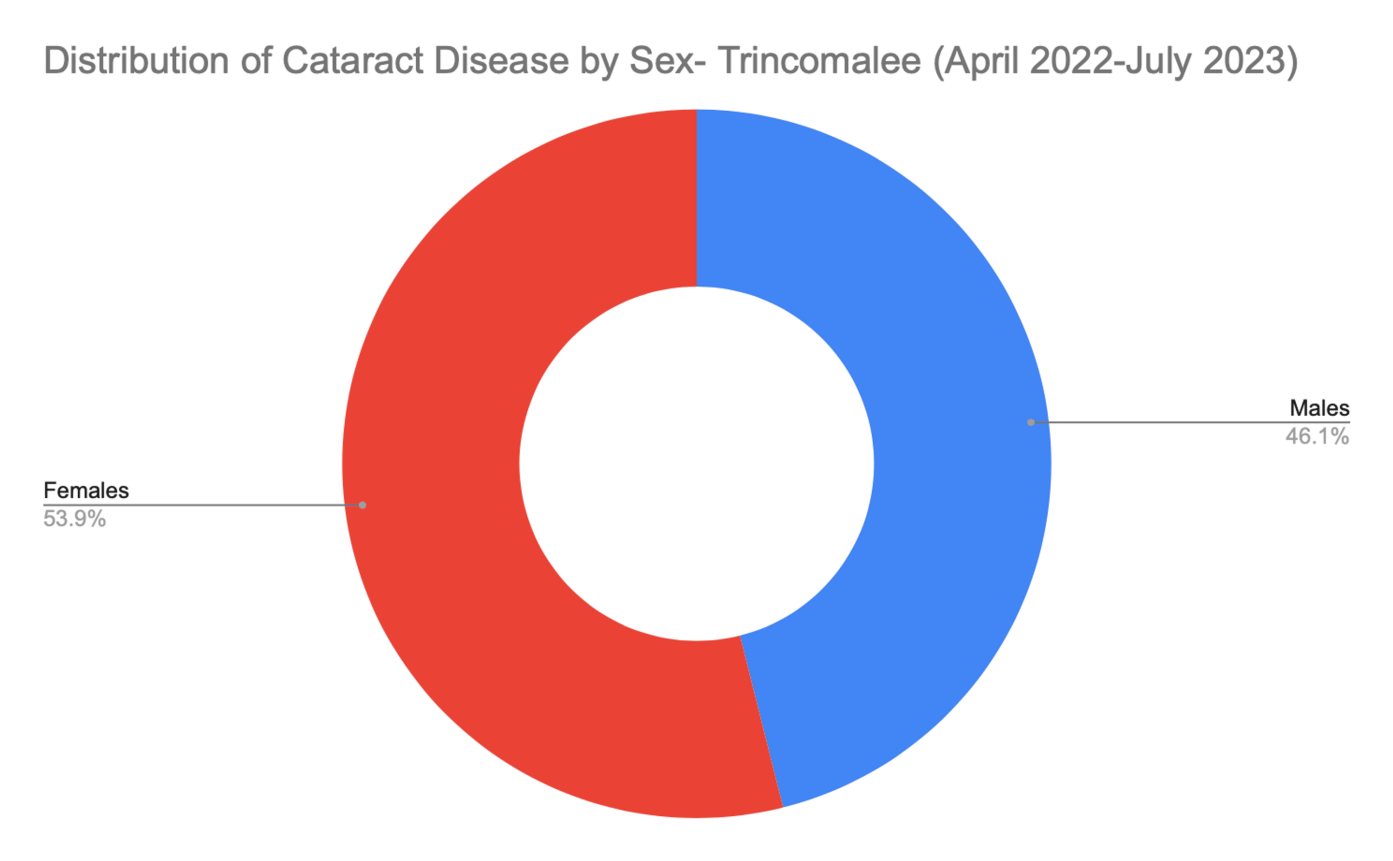
Distribution of patients with cataracts (by sex) in Trincomalee district (April 2022 to July 2023)

**Figure 2:**
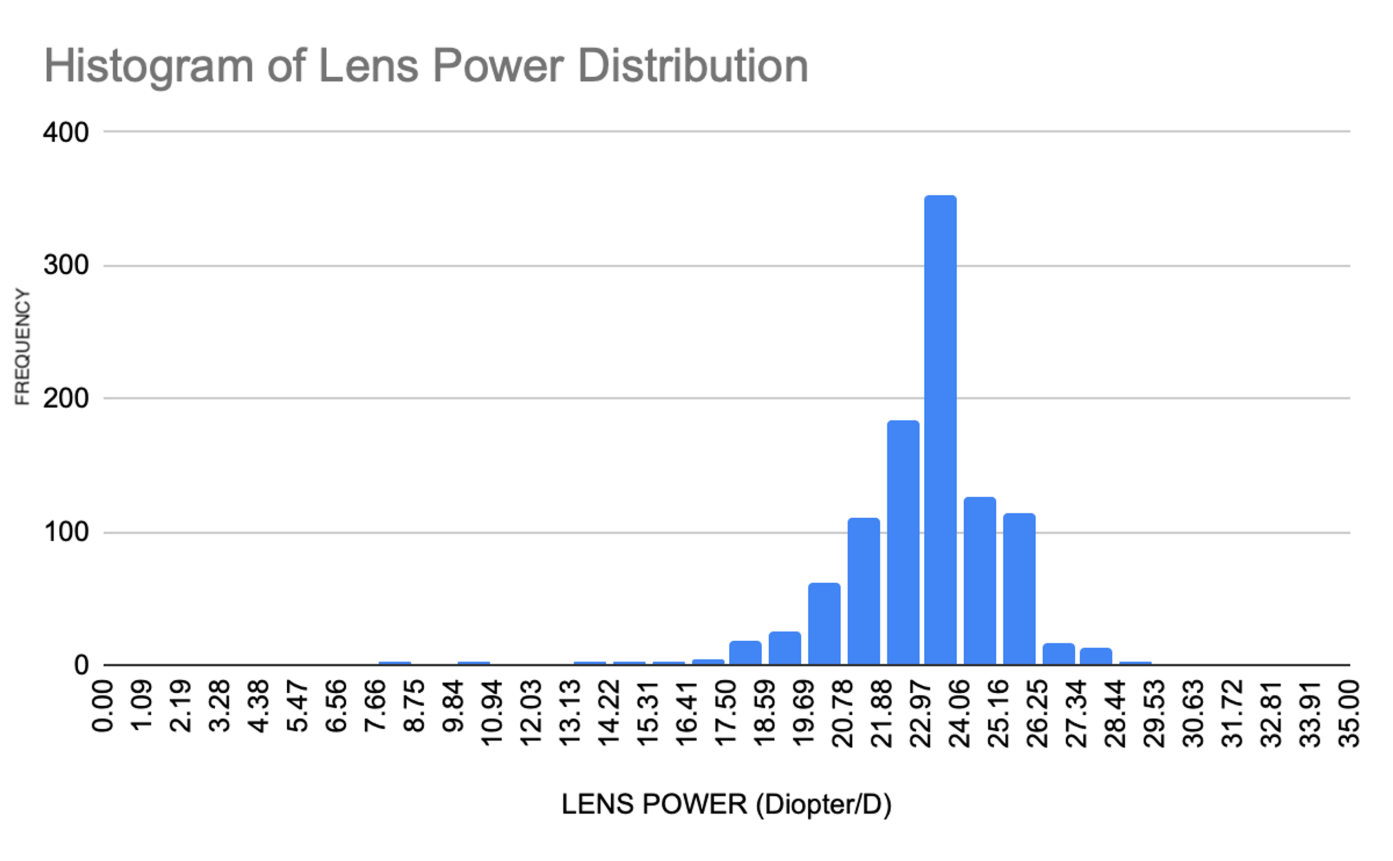
Lens power distribution of patients with cataracts in Trincomalee district (April 2022 to July 2023)

The cohort of 1,045 patients comprised 476 male (46%) and 557 female (53%) patients (Figure 1), indicating a higher prevalence of cataract surgeries among females. Figure 2 shows the distribution of lens powers (in diopters [D]) for the cohort. The mean was 22.95 D, with the majority of patients requiring lenses between 20.0 and 25.0 D. The peak frequency occurred at approximately 22.97 D.

Sex differences in lens power

As shown in Table 1, the IOLs that the patients received had a power that ranged from 3 to 30 D. The mean ± SD lens power across the entire cohort was 22.95 ± 2.398 D. By sex, the mean ± SD lens power was 22.75 ± 2.250 D for the male patients and 23.11 ± 2.519 D for the female patients, a difference of 0.36 D (95% CI 0.064-0.653). There was a significant difference in the mean lens power between the sexes (independent-samples t-test, p = 0.0168), suggesting that, on average, female patients required slightly stronger lenses compared with male patients in this cohort.

**Table 1:** Details on the intraocular lenses used and comparison between the sexes for patients with cataracts in Trincomalee district (April 2022 to July 2023). The unpaired t-test was used to calculate the t-value and p-value for the comparison between males and females.

Measure	Parameter	Value
Intraocular lens power	Range (D)	3-30
	Mean ± SD (D)	22.95 ± 2.398
Lens power by sex	Male, mean ± SD (D)	22.75 ± 2.250
	Female, mean ± SD (D)	23.11 ± 2.519
Statistical analysis	t-value	2.395
	p-value	0.0168

Age distribution

**Figure 3:**
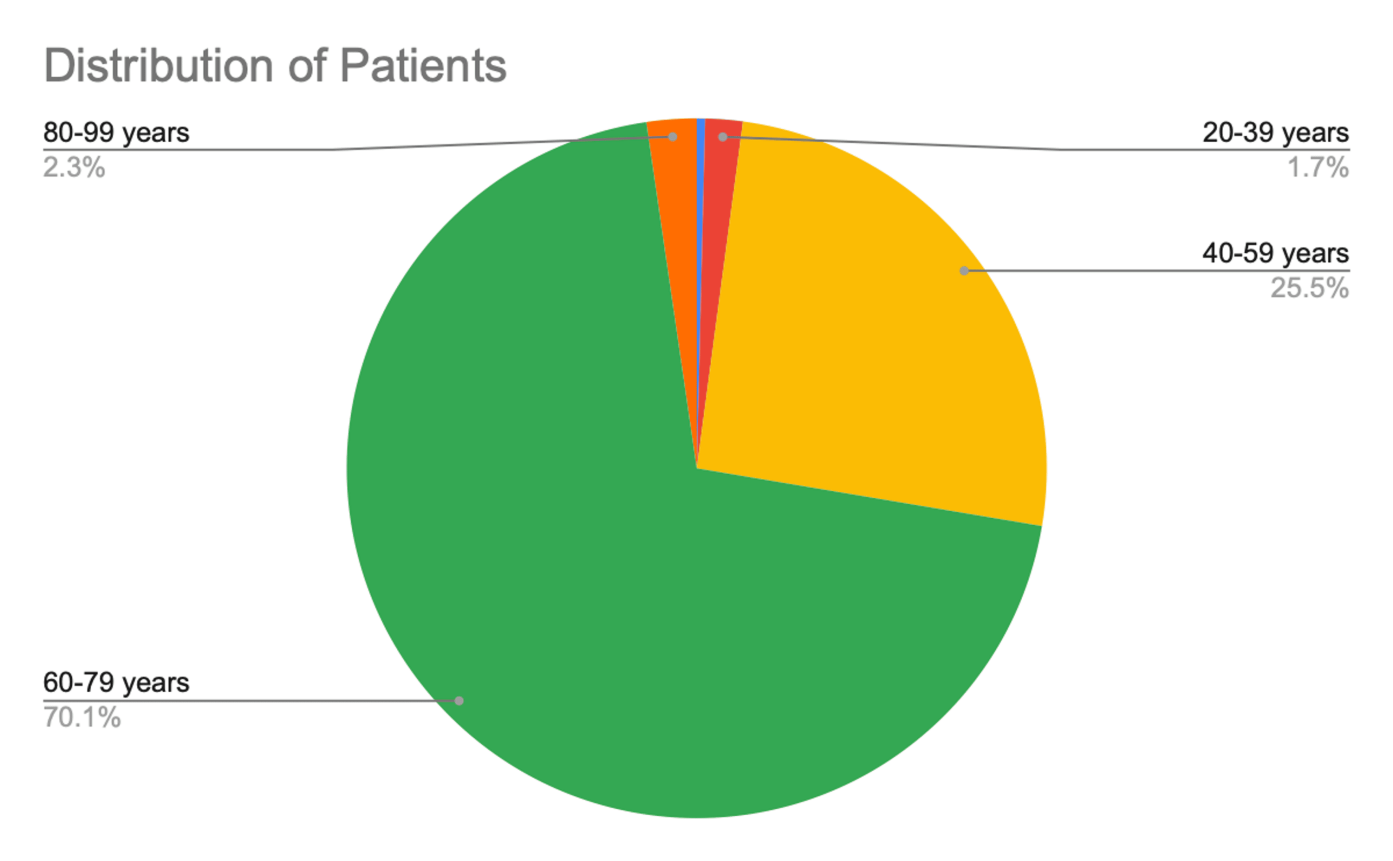
The distribution of patients with cataracts (by age group) in Trincomalee district (April 2022 to July 2023)

Figure 3 shows the distribution of the patients with cataracts by age. We observed the highest prevalence of cataracts in patients aged 60-79 years. Table 2 shows the mean ± SD lens power for each age group.

**Table 2:** The lens power and number of patients with cataracts for each age group in Trincomalee district (April 2022 to July 2023) *Due to illegible handwriting or missing documentation, likely resulting from human error, some records had to be from excluded age-related analyses (three patients).

Age group (years)	Lens power (D); Mean ± standard deviation	Number of patients
0-19	19.63 ± 2.016	4
20-39	21.58 (±5.386)	18
40-59	23.12 ± 2.491	266
60-79	22.94 ± 2.247	730
80-99	23.15 ± 1.697	24
	Total*	1042

One-way ANOVA revealed a significant difference in the mean lens power between at least two of the age groups (F = 3.8126; p = 0.0044). Table 3 shows the significant differences between the age groups. Overall, the <19-year-old age group had a significantly lower mean lens power compared with three of the other age groups (40-59, 60-79, and 80-99 years). Finally, Table 4 shows the mean lens power and number of patients by age group and sex.

**Table 3:** Results of Tukey’s post hoc comparisons for mean diopter differences across age groups. The overall analysis of variance (ANOVA) was performed to assess differences in mean diopter measurements between age groups, yielding an F-value of 3.812623 (p = 0.004392). Significant pairwise comparisons were made using Tukey’s post hoc test.

Significant Age Group Comparison (years)	Mean Diopter Difference (D)	Tukey Q-Value	p-Value
Group 1 (<19) vs. Group 3 (40-59)	3.4972	4.1169	0.03015
Group 1 (<19) vs. Group 4 (60-79)	3.3134	3.919	0.04496
Group 1 (<19) vs. Group 5 (80-99)	3.5208	3.8661	0.04984

**Table 4:** The mean lens power of patients with cataracts (by age group and sex) in Trincomalee district (April 2022 to July 2023) Due to illegible handwriting or missing documentation, likely resulting from human error, some records had to be excluded from the sex-related (12 patients) and age-related (three patients) analyses.

Age group (years)	Sex	Number of patients	Mean ± standard deviation lens power (D)
0-19	Female	2	18.25 ± 0.354
	Male	2	21.00 ± 2.121
20-39	Female	7	23.86 ± 2.193
	Male	11	20.14 ± 6.364
40-59	Female	158	23.07 ± 2.875
	Male	104	23.22 ± 1.813
60-79	Female	380	23.15 ± 2.358
	Male	345	22.69 ± 2.104
80-99	Female	10	22.90 ± 2.145
	Male	14	23.32 ± 1.353

## Discussion

We conducted this study amid a significant economic crisis in Sri Lanka. The availability of essential medical supplies, including IOLs, was severely affected. Our primary aim was to form a scientific basis to guide IOL donations, by determining the average lens power and its distribution by age and sex in patients with cataracts in Trincomalee district. The average lens power among patients who underwent cataract surgery was 22.95 D. This information is crucial for surgical planning in resource-constrained settings, where accurate prediction of lens requirements can optimize the use of donated supplies. The patients received IOLs with a power of 3-30 D, highlighting the variability in patient needs, and the importance of a diverse inventory of lenses to accommodate these differences.

According to the 2019 Global Burden of Disease Study, the global prevalence of cataracts by sex is 46% for men and 54% for women [6]. Our cohort showed a comparable distribution, with a higher prevalence of cataracts in female patients compared with male patients. Globally, women bear a disproportionate burden of cataract-related visual impairment, perhaps as a result of their longer life expectancy compared with men and other biological factors.

In addition to the sex difference regarding the prevalence of cataracts, the female patients in our cohort had a significantly larger mean lens power compared with the male patients. In our literature review, we found a study from Southwestern Ethiopia in which the authors used optical biometry and recorded a significantly higher mean lens power in female patients compared with male patients [7]. This sex difference suggests potential anatomical or physiological variations that could influence lens power requirements. Understanding these differences is essential to tailor lens procurement strategies to ensure that both male and female patients receive optimal care. This may warrant considering a patient’s sex when using ultrasound biometry to determine the ideal lens power.

The mean lens power varied between the age groups, with the youngest group (0-19 years) having the lowest lens power (19.63 D) and the oldest group (80-99 years) having the highest lens power (23.15 D). We noted significant differences between the youngest age group and other age groups. These findings emphasize the necessity for age-specific considerations in lens selection and inventory management.

The mean lens power for our patients in Trincomalee eistrict (22.95 D) is notably higher compared with what has been reported in Western and African populations: 20.72 D in Caucasian populations, 20.61 D in Pacific populations [8], 19.34 D in Eastern Ethiopia [9], 19.25 ± 1.8 D in Nigeria [10], 21.08 ± 7.36 D in India [11], 21.2 ± 2.35 D in Pakistan [12], and 21.60 ± 1.74 D in Nepal [13]. These regional differences warrant further investigation to enhance the understanding of cataract development and progression in diverse populations.

Despite the valuable insights we have gained, this study has several limitations. The retrospective design and reliance on existing medical records may have introduced selection bias and thus limited the generalizability of our findings. Additionally, we conducted the study at a single centre, and the results may not fully represent other regions of Sri Lanka. Additional research with larger, multi-centre studies is needed to confirm these findings and explore the underlying causes of observed differences in diopter sizes.

## Conclusions

The implications of this study are significant for the healthcare system in Trincomalee district and similar resource-limited settings. By providing a scientific basis for predicting IOL requirements, our study supports more effective and efficient surgical inventory planning, ensuring that patients receive timely and appropriate surgical interventions. Moreover, the data can inform future humanitarian efforts, guiding donors to provide lenses that match the specific needs of the population. Our findings highlight important sex- and age-related differences, which offer a valuable resource for surgical planning and lens donation strategies to ultimately improve cataract care in resource-limited settings. Continued monitoring and research are necessary to adapt to changing demographics and healthcare challenges, ensuring that all patients receive the best possible care.
